# Assessment of lamina cribrosa curvature in thyroid orbitopathy: a cross-sectional optical coherence tomography study

**DOI:** 10.1186/s12886-026-05473-6

**Published:** 2026-10-23

**Authors:** Gülistan Oyur, İdil Çelen Arabacı

**Affiliations:** 1 Department of Ophthalmology, University of Health Sciences, Başakşehir Çam and Sakura City Hospital, Istanbul, Türkiye; 2 Department of Ophthalmology, Yüksekova State Hospital, Hakkari, Türkiye

**Keywords:** Thyroid orbitopathy, Lamina cribrosa, Lamina cribrosa curvature index, Optical coherence tomography

## Abstract

**Background:**

To evaluate lamina cribrosa (LC) morphology using optical coherence tomography (OCT) in patients with thyroid orbitopathy (TO) and to investigate whether lamina cribrosa curvature (LCC) parameters, particularly the lamina cribrosa curvature index (LCCI), are associated with disease activity or the presence of TO.

**Methods:**

This cross-sectional study included 92 participants: 12 with active TO, 40 with inactive TO, and 40 healthy controls. Right and left eyes were analyzed separately. Optic nerve head imaging was performed using swept-source optical coherence tomography. Lamina cribrosa thickness, depth, curvature width, curvature depth, and LCCI were compared among active TO, inactive TO, and control groups. Active and inactive TO patients were also combined for case–control analysis.

**Results:**

There were no significant differences among the three groups in terms of sex distribution or axial length (*p* > 0.05). Hertel exophthalmometry (HE) measurements and intraocular pressure were significantly higher in the TO groups compared with controls (*p* < 0.001). Regarding LC morphology, no statistically significant differences were observed among active TO, inactive TO, and healthy controls in LCT, LCD, LCCW, LCCD, or LCCI for either right or left eyes. Similarly, case–control analysis comparing the combined TO group with healthy controls demonstrated no significant differences in any LC parameter (all *p* > 0.05). A direct pairwise comparison between active and inactive TO likewise showed no statistically significant difference in any LC parameter.

**Conclusions:**

LC curvature and related structural parameters did not show statistically significant differences between patients with TO and healthy individuals. These findings suggest that, in patients with TO without dysthyroid optic neuropathy, LCCI alone may have limited utility as a structural marker of disease presence or clinical activity.

## Background

Thyroid orbitopathy (TO) is an autoimmune inflammatory disorder affecting orbital tissues, often seen in patients with Graves’ disease [1]. The condition is characterized by enlargement of extraocular muscles, expansion of orbital adipose tissue, and increased orbital connective tissue volume, leading to clinical manifestations such as eyelid retraction, proptosis, diplopia, and, in severe cases, dysthyroid optic neuropathy (DON) [2]. These orbital changes may exert mechanical stress on intraorbital structures, including the optic nerve, even in the absence of clinically overt optic neuropathy [3].

Optic nerve involvement in TO has traditionally been evaluated using functional tests and clinical signs; however, structural alterations may precede detectable functional impairment [4]. Optical coherence tomography (OCT) has therefore gained increasing importance as a noninvasive tool for assessing subclinical structural changes in the optic nerve head and peripapillary retina [5]. Previous OCT studies in TO have primarily focused on retinal nerve fiber layer and ganglion cell layer measurements, with inconsistent findings reported across different disease stages [2, 6].

The lamina cribrosa (LC) is a key biomechanical component of the optic nerve head, consisting of a collagenous meshwork through which retinal ganglion cell axons exit the globe [7]. It plays a critical role in maintaining axonal integrity by providing structural support and modulating mechanical forces acting on the optic nerve [7, 8]. Alterations in LC morphology have been implicated in various optic neuropathies, particularly glaucoma [7–9].

Advances in enhanced depth imaging and swept-source OCT technologies have enabled in vivo visualization and quantitative assessment of LC morphology [10]. Parameters such as LCT and LCD have been widely investigated; however, these measurements may be influenced by interindividual anatomical variability, including optic disc size and reference plane selection [11]. To address these limitations, the lamina cribrosa curvature index (LCCI) has been introduced as a dimensionless parameter that quantifies the posterior bowing of the LC relative to its width, providing a standardized assessment of lamina cribrosa curvature (LCC) that is less dependent on disc size [11, 12].

LCCI has been extensively studied in glaucomatous optic neuropathy and has been shown to reflect LC deformation associated with mechanical stress at the optic nerve head [13]. More recently, LC-based parameters have been explored in non-glaucomatous conditions, suggesting that LC morphology may be altered in disease states characterized by chronic orbital or intracranial pressure changes [10, 14, 15]. Nevertheless, data regarding LC curvature in TO remain limited.

In particular, the relationship between LC morphology and disease activity in TO has not been fully clarified [10].Although recent studies have evaluated LC parameters, including the LCCI, in patients with TO, reported findings have been variable and have primarily focused on comparisons between TO and healthy controls [14, 16]. Data specifically examining potential differences between active and inactive disease stages remain limited. Given that active and inactive TO differ substantially in inflammatory burden and orbital tissue dynamics, it remains uncertain whether these distinctions are reflected in LC morphology on OCT [10, 14, 17]. Therefore, the present study aimed to evaluate LCC parameters in patients with TO and healthy controls and to assess LC morphology across active and inactive clinical stages using OCT.

## Methods

### Study design and ethics approval

This cross-sectional observational study was conducted at a tertiary referral center in accordance with the principles of the Declaration of Helsinki. The study protocol was approved by the local institutional ethics committee, and written informed consent was obtained from all participants prior to inclusion.

### Study population

A total of 92 participants were included in the study and classified into three groups: active TO (*n* = 12), inactive TO (*n* = 40), and healthy controls (*n* = 40). Both eyes were evaluated, and right and left eye data were analyzed separately to account for inter-eye correlation.

### Clinical evaluation and group classification

The diagnosis of TO was established based on clinical findings and thyroid function tests. Disease activity was assessed using the Clinical Activity Score (CAS) [18]. Patients with CAS ≥ 3 were classified as having active TO, while those with CAS < 3 were classified as having inactive TO, both of moderate severity according to the EUGOGO criteria.

### Clinical evaluation

Intraocular pressure was measured using Goldmann applanation tonometry (Haag-Streit AG, Köniz, Switzerland) on the same day as OCT imaging. Proptosis was assessed with HE (Inami & Co., Ltd., Tokyo, Japan). Axial length measurements were obtained using optical biometry (IOLMaster 700, Carl Zeiss Meditec AG, Jena, Germany). Patients with a previous diagnosis of glaucoma or ocular hypertension, optic neuropathy other than thyroid-related disease, previous intraocular surgery (except uncomplicated cataract surgery), high myopia, or poor-quality OCT images were excluded. Elevated IOP values detected during the study examination were recorded as part of the clinical profile of thyroid orbitopathy, provided that glaucomatous optic neuropathy was absent. None of the included participants had a diagnosis of dysthyroid optic neuropathy.

### OCT imaging

Optic nerve head imaging was performed using swept-source optical coherence tomography angiography (SS-OCTA) (DRI OCT Triton; Topcon Corporation, Tokyo, Japan). Structural B-scan images acquired during OCT angiography scanning were used for LC evaluation.

Horizontal B-scan images passing through the center of the optic disc were selected for analysis. A single central horizontal B-scan was used, consistent with the original LCCI methodology and with several prior OCT-based studies of lamina cribrosa morphology in thyroid orbitopathy, as it provides a standardized, reproducible reference section through the widest point of the optic disc cup; image quality was assessed using the device-reported Image Quality (IQ) index (DRI OCT Triton, Topcon Corporation, scale 0-100), and scans with an IQ below 40 were excluded. Scans with poor image quality, motion artifacts, segmentation errors, or insufficient visualization of the LC were excluded from further analysis. Of the 86 clinically eligible TO patients who underwent OCT imaging, 34 were excluded for inadequate OCT image quality, yielding the final analyzed samples of 12 active TO and 40 inactive TO patients. LC boundaries and curvature parameters were manually delineated using the caliper tool within the device’s native viewer software (IMAGEnet 6, Topcon Corporation).

### LC measurements

Horizontal B-scan images passing through the center of the optic disc were analyzed. The Bruch’s membrane opening (BMO) was identified, and a reference line was constructed by connecting the nasal and temporal BMO points.

#### LC parameters were measured as follows

Lamina cribrosa thickness (LCT): distance between the anterior and posterior borders of the lamina cribrosa.

Lamina cribrosa depth (LCD): perpendicular distance from the BMO reference line to the anterior LC surface at the midpoint.

Lamina cribrosa curvature width (LCCW): horizontal distance between the two points on the anterior LC surface defining the curvature span.

lamina cribrosa curvature depth (LCCD): maximum perpendicular distance from the midpoint of the LCCW to the anterior LC surface.

LCCI: was calculated as:

LCCI= (LCCD /LCCW)×100.

All linear measurements (LCT, LCD, LCCW, and LCCD) were expressed in micrometers (µm). LCCI is a dimensionless ratio, conventionally expressed as a percentage-like index.

**Fig. 1:**
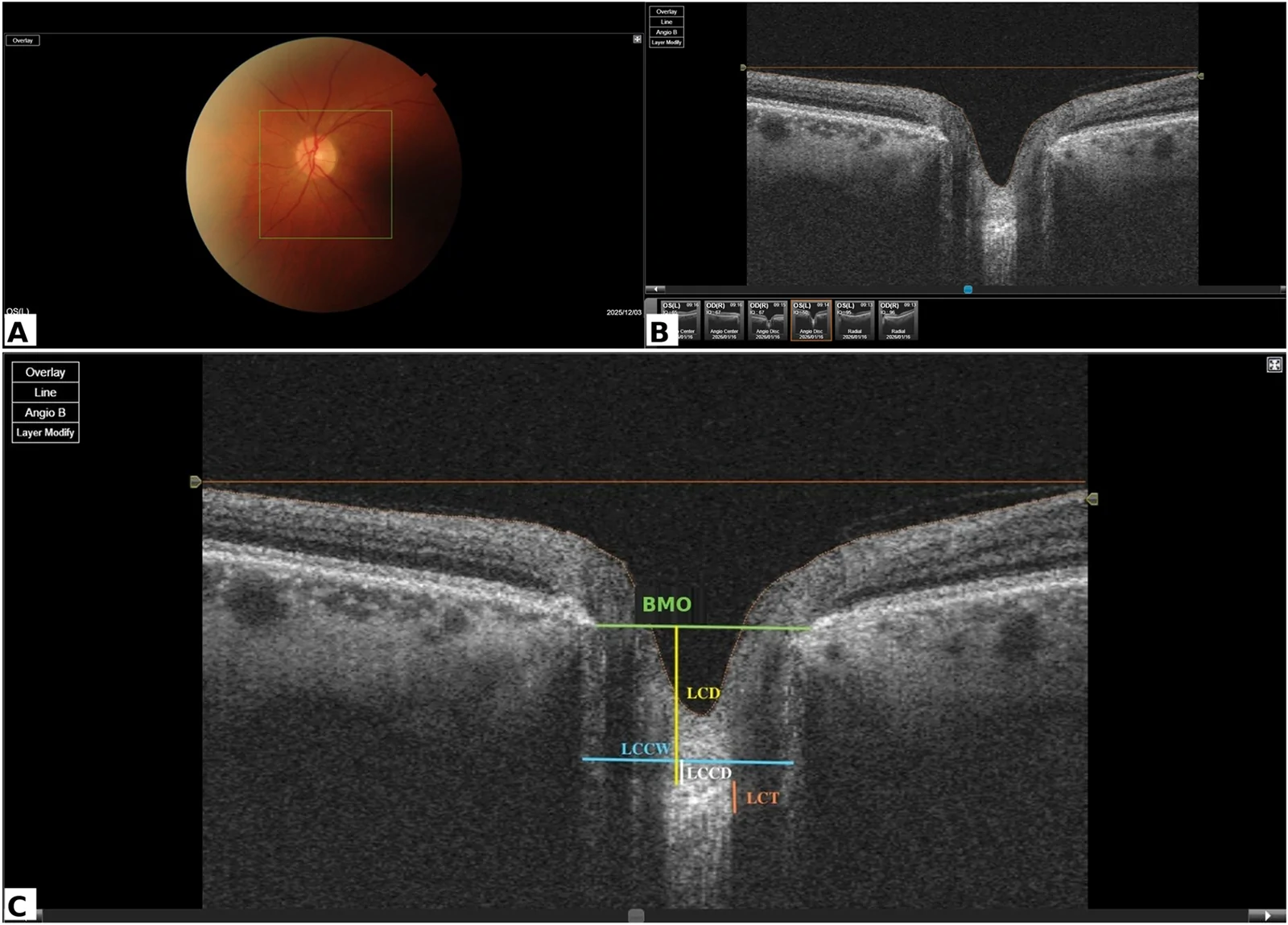
**A**: Fundus photograph of the optic disc. Fundus photograph of the left eye showing a clinically normal-appearing optic disc. The green square indicates the scanning area used for swept-source optical coherence tomography angiography. **B**: Structural B-scan of the optic nerve head obtained by SS-OCTA. Representative structural B-scan of the optic nerve head obtained using swept-source optical coherence tomography angiography. The lamina cribrosa is visualized as a hyperreflective structure beneath the prelaminar tissue. **C**: Measurement of lamina cribrosa curvature parameters. Annotated swept-source optical coherence tomography image. The Bruch’s membrane opening (BMO) points were identified, and a reference line connecting the two BMO points (green line) was used as the baseline. Lamina cribrosa depth (LCD) was defined as the perpendicular distance from the BMO reference line to the anterior surface of the lamina cribrosa at the midpoint between the two BMO reference points (yellow line), distinct from LCCD (below), which represents the maximum posterior displacement within the curvature span. Lamina cribrosa curvature width (LCCW) (blue line) represents the horizontal distance between the two reference points at the level of the anterior lamina cribrosa surface. Lamina cribrosa curvature depth (LCCD) indicates the maximum posterior displacement of the lamina cribrosa relative to the reference line. Lamina cribrosa thickness (LCT) (orange line) was measured at the central point of the lamina cribrosa. The lamina cribrosa curvature index (LCCI) was calculated as: LCCI = (LCCD / LCCW) × 100

LC parameters were measured as described above and illustrated in Fig. 1.

All measurements were performed by a single masked observer to minimize measurement bias.

### Statistical analysis

Mean, standard deviation, median, minimum, maximum value frequency and percentage were used for descriptive statistics. The distribution of variables is measured by Kolmogorov-Smirnov and Shapiro-Wilk tests. Normality was assessed independently for each variable and for each eye; consequently, the parametric or non-parametric test selected for a given clinical or LC parameter could differ between right and left eyes when their underlying distributions differed. ANOVA (Tukey test) and independent samples t test were used to analyze independent quantitative data with normal distribution. Kruskal-Wallis and Mann-Whitney U tests were used in the analysis of independent quantitative data with non-normal distribution. Chi-Square test was used for the comparison of the qualitative data. Associations between LC parameters and Hertel exophthalmometry or intraocular pressure were evaluated using Spearman correlation analysis across the entire study population (all 92 participants, including controls, both eyes pooled); this exploratory analysis was not corrected for multiple comparisons, and results should be interpreted accordingly. SPSS 27.0 was used for statistical analyses. Because both eyes of each participant were evaluated, right and left eye data were analyzed separately rather than pooled as independent observations, thereby avoiding artificial inflation of the sample size due to inter-eye correlation.

**Table 1:** Overall demographic, clinical, and lamina cribrosa characteristics of the study population (n = 92)

		Min-Max		Median	Mean±sd/n-%	
Age		18	- 71	44.5	42.9	± 12.3
Sex	Male				34	37.00%
	Female				58	63.00%
Inactive /Active/ Control	Inactive				40	43.50%
	Active				12	13.00%
	Control				40	43.50%
**HE**						
Right		14	- 30	18	19.6	± 3.9
Left		14	- 30	18.5	19.8	± 3.9
**IOP**						
Right		10	- 35	16	16.6	± 4.2
Left		8	- 35	16	16.6	± 4.4
**AL**						
Right		21.4	- 24.7	23.3	23.3	± 0.5
Left		21.5	- 24.3	23.3	23.3	± 0.5
**LCT**						
Right		208	- 361	260	265.1	± 37.2
Left		172	- 422	256.5	261.4	± 41.5
**LCD**						
Right		276	- 878	480	503	± 110.7
Left		247	- 950	468.5	492	± 119
**LCCW **						
Right		1117	- 1927	1538.5	1530	± 143.4
Left		1184	- 1970	1523	1529.7	± 161.2
**LCCD**						
Right		122	- 375	185.5	193.6	± 39.9
Left		110	- 247	181.5	184.5	± 28.2
**LCCI**						
Right		8.5	- 23.5	12.2	12.7	± 2.5
Left		8.3	- 16.9	12	12.1	± 1.9

Values are presented as mean ± standard deviation (SD), median (minimum–maximum),or number (%), as appropriate. Ocular parameters are presented separately for right and left eyes.HE: Hertel exophthalmometry; IOP: intraocular pressure; AL: axial length; LCT: lamina cribrosathickness; LCD: lamina cribrosa depth; LCCW: lamina cribrosa curvature width; LCCD: laminacribrosa curvature depth; LCCI: lamina cribrosa curvature index

Because age differed significantly among groups, all primary between-group comparisons of LC parameters were repeated using analysis of covariance (ANCOVA) adjusting for age and axial length, performed separately for each eye. To provide a primary analysis that incorporates both eyes while accounting for within-subject (inter-eye) correlation, generalized estimating equations (GEE) with an exchangeable working correlation structure and subject-level clustering were additionally fitted for each LC parameter, adjusting for age. A direct pairwise comparison between the active TO and inactive TO groups was performed using the independent-samples t-test (or Mann-Whitney U test, as appropriate) and repeated using age-adjusted linear models, with mean differences and 95% confidence intervals reported. For the correlation analysis between LC parameters and Hertel exophthalmometry/intraocular pressure (Table 5), right and left eyes were pooled as a combined dataset; to account for the resulting within-subject clustering, these associations were additionally examined using GEE models with subject-level clustering, and the corresponding cluster-adjusted p-values are reported alongside the naive (unclustered) Spearman correlation coefficients. 

**Table 2:** Age, sex, and smoking status stratified by group (active TO, inactive TO, control). Group-level comparisons of Hertel exophthalmometry, intraocular pressure, and axial length are presented in Table 3

Characteristic	Active TO (n = 12)	Inactive TO (n = 40)	Control (n = 40)	*p*-value	Test
Age (years), mean ± SD	51.4 ± 8.2	41.3 ± 12.7	42.0 ± 12.2	0.042	K-W
Sex, male/female, n	8 / 4	13 / 27	13 / 27	0.073	X²
Smoking, yes, n (%)	6 (50.0)	25 (62.5)	0 (0.0)	<0.001	X²

K-W: Kruskal-Wallis test; X²: chi-square test. TO: thyroid orbitopathy

**Table 3:** Comparison of clinical and lamina cribrosa parameters among inactive thyroid orbitopathy, active thyroid orbitopathy, and healthy control groups

		¹Inactive (*n*:40)	Group	²Active (*n*:12)	Group	³Control Group (*n*:40)		*p*
***HE***								
Right	Mean ± sd	21.9	± 3.3	22.3	± 4.3	16.5	± 1.4	***< 0.001*** ^K^
	Median	21.0		22.5		16.0¹²		
Left	Mean ± sd	21.9	± 3.2	23.5	± 3.6	16.6	± 1.3	***< 0.001*** ^K^
	Median	22.0		25.0		16.0¹²		
***IOP***								
Right	Mean ± sd	17.6	± 3.3	21.7	± 5.8	14.0	± 2.4	***< 0.001*** ^K^
	Median	18.0¹		21.0		14.0¹²		
Left	Mean ± sd	17.4	± 3.3	22.8	± 5.6	14.0	± 2.5	***< 0.001*** ^K^
	Median	18.0¹		22.0		14.0¹²		
***AL***								
Right	Mean ± sd	23.2	± 0.6	23.1	± 0.7	23.4	± 0.4	0.273^K^
	Median	23.3		23.2		23.4		
Left	Mean ± sd	23.2	± 0.5	23.2	± 0.6	23.4	± 0.4	0.216^A^
	Median	23.3		23.2		23.4		
***LCT***								
Right	Mean ± sd	264.7	± 41.2	247.7	± 37.6	270.7	± 31.8	0.077^K^
	Median	259.0		229.5		265.5		
Left	Mean ± sd	255.1	± 37.5	256.4	± 28.0	269.2	± 47.8	0.292^A^
	Median	249.5		253.5		267.5		
***LCD***								
Right	Mean ± sd	522.4	± 133.1	494.0	± 83.9	486.2	± 90.4	0.652^K^
	Median	487.0		460.0		476.5		
Left	Mean ± sd	490.4	± 130.9	513.6	± 111.1	487.2	± 110.6	0.678^K^
	Median	449.0		498.0		480.0		
***LCCW***								
Right	Mean ± sd	1549.1	± 142.7	1504.2	± 132.8	1518.8	± 148.3	0.517^A^
	Median	1548.0		1483.5		1539.0		
Left	Mean ± sd	1520.8	± 164.8	1519.9	± 165.3	1541.6	± 159.6	0.828^A^
	Median	1492.5		1510.5		1537.0		
***LCCD***								
Right	Mean ± sd	197.5	± 42.4	186.9	± 18.4	191.8	± 42.3	0.675^K^
	Median	193.0		178.0		182.0		
Left	Mean ± sd	185.0	± 23.6	176.3	± 24.8	186.5	± 33.3	0.544^A^
	Median	184.0		178.5		180.5		
***LCCI***								
Right	Mean ± sd	12.8	± 2.7	12.5	± 1.4	12.6	± 2.7	0.910^K^
	Median	12.3		12.5		12.1		
Left	Mean ± sd	12.3	± 2.0	11.7	± 1.7	12.1	± 1.8	0.601^A^
	Median	12.2		11.6		12.2		

Values are presented as mean ± SD and median values. A: One-way ANOVA;K: Kruskal–Wallis test (with post hoc Mann–Whitney U test when appropriate)

¹ Indicates significant difference compared with the inactive group (p < 0.05)

² Indicates significant difference compared with the active group (p < 0.05).HE: Hertel exophthalmometry; IOP: intraocular pressure; AL: axial length;LCT: lamina cribrosa thickness; LCD: lamina cribrosa depth;LCCW: lamina cribrosa curvature width; LCCD: lamina cribrosa curvature depth;LCCI: lamina cribrosa curvature index

No a priori sample size calculation was performed, as published data on LCCI in thyroid orbitopathy were unavailable at the time of study design. To assess whether the current sample was adequate to detect a clinically meaningful difference, a post-hoc sensitivity analysis was conducted using the noncentral F-distribution for a one-way ANOVA (fixed-effects, omnibus, k = 3 groups; equivalent to G*Power’s “F tests – ANOVA: Fixed effects, omnibus, one-way” module). With the present total sample size (*N* = 92) and an alpha level of 0.05, a balanced-allocation calculation yielded approximately 55% power to detect a medium effect size (Cohen’s f = 0.25). Because the actual group sizes were unequal (active TO: *n* = 12; inactive TO: *n* = 40; controls: *n* = 40), the effective achieved power was lower still, approximately 30–45% depending on the assumed configuration of group means (harmonic-mean correction [19]; direct Monte Carlo simulation under the observed allocation). A total sample of approximately 157 participants (~ 52 per group) would be required to achieve 80% power for a medium effect under a balanced design with three groups. The study was therefore underpowered to reliably detect small-to-medium between-group differences, particularly for comparisons involving the small active-TO subgroup, and the observed effect sizes for LCCI were small. Accordingly, the absence of statistically significant differences in LC parameters should be interpreted as reflecting the precision limits of the present sample rather than as evidence against a true underlying biological effect.

## Results

### Demographic and clinical characteristics

A total of 92 participants were included in the study: 12 patients with active TO, 40 patients with inactive TO, and 40 healthy controls. Both eyes were evaluated, and right and left eye findings are presented separately.

Sex distribution did not differ significantly among the three groups (*p* = 0.073). Age showed a statistically significant difference (*p* = 0.042), with the active TO group being older than the inactive TO and control groups, while no significant difference was observed between inactive patients and controls.

HE values were significantly higher in both active and inactive TO groups compared with controls (*p* < 0.001). IOP values were also significantly elevated in the TO groups, with the highest values observed in the active TO group (*p* < 0.001). However, axial length did not differ significantly among the three groups (*p* > 0.05) (Table 1  and 3) 

### LC parameters: three-group comparison

LC structural parameters are summarized in Table 3.

There were no statistically significant differences among active TO, inactive TO, and control groups in any LC parameter.

LCT showed no statistically significant difference across groups (right eye *p* = 0.077; left eye *p* = 0.292).

LCD showed no statistically significant difference (right eye *p* = 0.652; left eye *p* = 0.678).

LCCW showed no statistically significant difference among groups (right eye *p* = 0.517; left eye *p* = 0.828).

LCCD showed no significant difference (right eye *p* = 0.675; left eye *p* = 0.544).

LCCI likewise showed no statistically significant difference among the three groups (right *p* = 0.910; left *p* = 0.601).

These findings remained essentially unchanged after adjustment for age and axial length (ANCOVA, all *p* > 0.11 for the omnibus three-group effect on any LC parameter) and after accounting for inter-eye correlation using GEE models with subject-level clustering (omnibus *p* = 0.68 for LCD, *p* = 0.59 for LCCW, *p* = 0.22 for LCCD, and *p* = 0.63 for LCCI). In the age-adjusted, eye-pooled GEE model, the omnibus group effect for LCT was borderline and not statistically significant (p = 0.055); however, the post-hoc pairwise contrast indicated thinner LCT in active TO than in control eyes (mean difference −22.5 μm, 95% CI −41.1 to −3.9). As this pairwise comparison was not prespecified and was not adjusted for multiple testing, it is reported as a nominally significant but unadjusted post-hoc difference rather than a confirmed finding (see Discussion). 

A direct pairwise comparison between the active TO and inactive TO groups showed no statistically significant difference in any LC parameter, either unadjusted (all *p* > 0.18) or after adjustment for age (all *p* > 0.25). Unadjusted mean differences (active minus inactive, 95% CI) were: LCT, -17.0 μm (-43.5 to 9.4) right eye and 1.3 μm (-19.4 to 22.0) left eye; LCD, -28.4 μm (-94.0 to 37.2) right eye and 23.2 μm (-56.2 to 102.6) left eye; LCCW, -44.9 μm (-137.9 to 48.1) right eye and − 0.9 μm (-115.0 to 113.3) left eye; LCCD, -10.5 μm (-27.8 to 6.7) right eye and − 8.7 μm (-25.7 to 8.3) left eye; and LCCI, -0.30 (-1.51 to 0.90) right eye and − 0.63 (-1.88 to 0.61) left eye. Age-adjusted mean differences (active minus inactive, 95% CI) were: LCT, -19.4 μm (-48.1 to 9.3) right eye and − 1.9 μm (-27.1 to 23.3) left eye; LCD, -39.2 μm (-127.0 to 48.6) right eye and 16.2 μm (-73.8 to 106.3) left eye; LCCW, -63.3 μm (-162.1 to 35.6) right eye and − 17.5 μm (-134.1 to 99.1) left eye; LCCD, -15.6 μm (-42.7 to 11.4) right eye and − 9.4 μm (-26.4 to 7.6) left eye; and LCCI, -0.49 (-2.24 to 1.26) right eye and − 0.51 (-1.91 to 0.90) left eye. All confidence intervals, unadjusted and age-adjusted, were wide and included zero, consistent with the limited precision of the active-TO subgroup discussed above.

### Case–control analysis

When active and inactive patients were combined into a single thyroid orbitopathy group and compared with healthy controls, no statistically significant differences were observed in any LC parameter (all *p* > 0.05) (Table 4).

**Table 4:** Comparison of demographic, clinical, and lamina cribrosa parameters between thyroid orbitopathy (case) and control groups

		Control Group (*n*:40)			Case Group (*n*:52)			*p*
		Mean ± sd/*n*-%		Median	Mean ± sd/*n*-%		Median	
Age		42.1	± 12.2	43.5	43.6	± 12.5	45.5	0.670^m^
Sex	Male	13	32.5%		21	40.4%		0.437^X²^
	Female	27	67.5%		31	59.6%		
***HE***								
Right		16.5	± 1.4	16.0	22.0	± 3.5	21.0	***< 0.001*** ^m^
Left		16.6	± 1.3	16.0	22.2	± 3.3	22.5	***< 0.001*** ^m^
***IOP***								
Right		14.0	± 2.4	14.0	18.6	± 4.3	18.0	***< 0.001*** ^m^
Left		14.0	± 2.5	14.0	18.6	± 4.5	18.0	***< 0.001*** ^m^
***Axial***								
Right		23.4	± 0.4	23.4	23.2	± 0.6	23.3	0.146^m^
Left		23.4	± 0.4	23.4	23.2	± 0.5	23.3	0.093^t^
***LCT***								
Right		270.7	± 31.8	265.5	260.8	± 40.7	254.5	0.084^m^
Left		269.2	± 47.8	267.5	255.4	± 35.3	250.0	0.116^t^
***LCD***								
Right		486.2	± 90.4	476.5	515.9	±123.4	486.0	0.436^m^
Left		487.2	±110.6	480.0	495.7	±126.0	455.0	0.872^m^
***LCCW***								
Right		1518.8	±148.3	1539.0	1538.7	±140.5	1534.0	0.512^t^
Left		1541.6	±159.6	1537.0	1520.6	±163.3	1492.5	0.539^t^
***LCCD***								
Right		191.8	± 42.3	182.0	195.0	± 38.3	190.0	0.478^m^
Left		186.5	± 33.3	180.5	183.0	± 23.9	182.0	0.554^t^
***LCCI***								
Right		12.6	± 2.7	12.1	12.7	± 2.4	12.3	0.671^m^
Left		12.1	± 1.8	12.2	12.2	± 2.0	12.0	0.892^t^

Footnote: Values are expressed as mean ± SD or number (%). t: Independent samples t-test; m: Mann–Whitney U test; X²: Chi-square test (Fisher’s exact test when appropriate).HE: Hertel exophthalmometry; IOP: intraocular pressure; AL: axial length;LCT: lamina cribrosa thickness; LCD: lamina cribrosa depth;LCCW: lamina cribrosa curvature width; LCCD: lamina cribrosa curvature depth;LCCI: lamina cribrosa curvature index

### Correlation analysis

Exploratory Spearman correlation analysis between LC parameters and Hertel exophthalmometry/intraocular pressure is summarized in Table 5. Of ten correlations tested, only LCD showed a nominally significant association with Hertel exophthalmometry (*r* = 0.151, *p* = 0.041); however, this association was no longer statistically significant after accounting for within-subject clustering between eyes (GEE, *p* = 0.100) and did not survive correction for multiple comparisons. No other LC parameter showed a significant association with Hertel exophthalmometry or intraocular pressure.

**Table 5:** Correlation analysis between lamina cribrosa parameters and Hertel exophthalmometry and intraocular pressure

	Hertel		IOP	
	*r*	*p*	*r*	*p*
LCT	-0.123	0.096	-0.004	0.962
LCD	0.151	***0.041***	0.095	0.199
LCCW	-0.026	0.722	-0.032	0.666
LCCD	-0.014	0.851	0.019	0.801
LCCI	-0.004	0.962	0.052	0.487

Spearman correlation analysis

r: correlation coefficient; p: significance value. Correlations were calculated across the entire study population (all 92 participants, including controls). Right and left eyes were pooled as a combined dataset (n = 184 eye-level observations) for this exploratory analysis; because this approach does not account for within-subject (inter-eye) correlation, all associations were additionally examined using generalized estimating equation (GEE) models with subject-level clustering. Under this cluster-adjusted approach, the LCD-HE association was no longer statistically significant (standardized b = 0.126, 95% CI -0.024 to 0.276, p = 0.100), indicating that the nominally significant naive correlation (r = 0.151, p = 0.041) likely reflected the inflated eye-level sample size rather than a genuine bilateral association. As ten correlations were tested in total, this analysis should be considered exploratory rather than confirmatory; the nominal p = 0.041 does not survive correction for multiple comparisons (e.g., Bonferroni-adjusted a = 0.005)

HE: Hertel exophthalmometry; IOP: intraocular pressure; LCT: lamina cribrosa thickness; LCD: lamina cribrosa depth; LCCW: lamina cribrosa curvature width; LCCD: lamina cribrosa curvature depth; LCCI: lamina cribrosa curvature index

## Discussion

In this cross-sectional OCT study, we evaluated LC morphology in patients with active and inactive TO and compared these findings with healthy controls. We found that LCT, LCD, LCCW, LCCD, LCCI showed no statistically significant differences across groups according to either the presence of TO or disease activity status. These findings suggest that LC curvature, as quantified by LCCI, may not undergo substantial structural alteration in TO in the absence of overt dysthyroid optic neuropathy.

Seo et al. evaluated changes in LC depth in patients with TO undergoing orbital decompression, representing a subgroup with more pronounced orbital structural alterations [17]. In such cases, orbital crowding and apical compression may influence optic nerve head biomechanics and contribute to measurable changes in lamina configuration. In contrast, the present study excluded patients with overt DON, and most participants had clinically stable disease without evident compressive features. Under these conditions, the biomechanical forces acting on the LC may be insufficient to trigger detectable structural remodeling on OCT, at least within the resolution limits of current OCT imaging.

Romano et al. reported that LCT is significantly reduced in eyes with TO–related optic nerve compression compared with healthy controls and that a marked decrease in the perineural subarachnoid space increases the translaminar pressure gradient, leading to LC deformation and ganglion cell complex damage [20]. In contrast, the present study included only TO patients without dysthyroid optic neuropathy, and no significant differences were detected in LCT or curvature-based LC parameters (LCCD, LCCW, LCCI) between either active or inactive TO and healthy controls. This discrepancy suggests that pronounced LC remodeling may occur only when intraorbital/translaminar pressure and optic nerve compression reach a critical level, whereas LC structure is relatively preserved in milder TO or in the absence of neuropathy. Thus, Romano et al.’s findings imply that LC changes are characteristic of advanced, compressive disease, while the current results indicate that LC thickness and curvature are unlikely to serve as sensitive structural biomarkers of disease presence or activity in TO patients without optic neuropathy. Consequently, LC metrics in TO should be interpreted in the context of disease stage and the severity of optic nerve compression. Interestingly, in a post-hoc sensitivity analysis pooling both eyes, active TO showed nominally thinner LCT relative to controls (mean difference −22.5 μm, 95% CI −41.1 to −3.9; omnibus p = 0.055). Although this unadjusted post-hoc difference was not prespecified and was not corrected for multiple comparisons, its direction is consistent with Romano et al.‘s report of reduced LCT in TO-related optic nerve compression, and may warrant targeted evaluation of LCT specifically, as distinct from curvature-based parameters, in an adequately powered prospective cohort.

Ergen et al. reported significantly increased LCCI values in TO compared with healthy controls and interpreted LC curvature as a marker of optic nerve head deformation [14]. In contrast, in our cohort, LCCI values showed no statistically significant differences across active, inactive, and control groups, despite significantly higher Hertel exophthalmometry and intraocular pressure values in patients with thyroid orbitopathy. Although these clinical parameters were elevated in the TO groups, they were not accompanied by significant between-group differences in LCCI or other curvature-based LC parameters. These findings suggest that moderate orbital protrusion or intraocular pressure elevation in non-DON TO may be insufficient to produce detectable alterations in lamina cribrosa curvature. Differences in clinical severity, biomechanical load, or methodological approaches may therefore explain why increased LCCI values were observed in the study by Ergen et al., whereas LCC parameters remained stable in our cohort.

Ozer et al. evaluated LC parameters together with peripapillary vascular density in patients with TO and reported structural and microvascular alterations, particularly in the context of systemic corticosteroid treatment [16]. Their findings suggested that inflammatory and vascular mechanisms may contribute to optic nerve head changes during active disease. However, their cohort included patients undergoing immunosuppressive therapy, and LCC parameters were interpreted alongside dynamic vascular alterations rather than as isolated structural curvature metrics. In contrast, our study focused on curvature-based parameters and excluded patients with overt compressive neuropathy or acute therapeutic modulation. The absence of significant LCCI differences in our cohort may therefore reflect the relative structural stability of the LC in clinically stable or non-compressive disease, whereas inflammatory or treatment-related vascular changes observed by Ozer et al. may represent a different pathophysiological context.

Taken together, the discrepancies between our findings and those of Seo et al., Romano et al., Ergen et al., and Ozer et al. appear to converge on two common explanatory themes rather than reflecting unrelated, study-specific differences. First, disease severity and the presence of compressive optic neuropathy appear to be the principal determinant of detectable LC remodeling: studies reporting significant LC changes generally included patients with orbital decompression, overt optic nerve compression, or concurrent immunosuppressive treatment for active inflammation, whereas our cohort specifically excluded dysthyroid optic neuropathy and mainly comprised clinically stable disease. Second, methodological differences, including scan protocol, reference-plane definition, and whether curvature-based or thickness-based parameters were prioritized, likely contribute to between-study variability and limit direct comparability. Considered jointly, these observations suggest that LCC parameters may become informative primarily once compressive or inflammatory disease activity reaches a critical threshold, rather than serving as an early or continuous marker across the full spectrum of TO severity.

LC deformation has been most consistently demonstrated in glaucomatous optic neuropathy, where chronic intraocular pressure–related stress contributes to posterior laminar bowing and structural remodeling of the optic nerve head [21–23]. In this context, LC curvature alterations are thought to reflect sustained translaminar pressure gradients rather than transient pressure fluctuations. In TO, however, intraocular pressure elevations are often modest and may be influenced by increased episcleral venous pressure secondary to orbital congestion rather than primary aqueous outflow resistance [24, 25]. Consequently, the biomechanical load imposed on the LC may differ fundamentally from that observed in glaucoma. Furthermore, single-visit IOP measurements may not adequately capture the cumulative or chronic stress necessary to induce measurable LC remodeling, which may partly explain why elevated IOP in the TO groups was not accompanied by significant differences in LCCI. IOP was measured on the same day as OCT imaging in all participants, but only as a single time point; no data on IOP duration or prior IOP history were available, precluding assessment of cumulative pressure exposure. For this reason, IOP was not included as a covariate in our adjusted models: because IOP elevation in TO groups likely arises from orbital congestion secondary to the disease itself (e.g., via elevated episcleral venous pressure and reduced aqueous outflow), IOP plausibly lies on the causal pathway between TO and LC deformation rather than acting as an independent confounder, and adjusting for it risks removing part of the true biological effect rather than isolating a nuisance variable.

From a clinical perspective, our findings suggest that LCC parameters, including LCCI, may have limited utility as markers of disease activity in uncomplicated thyroid orbitopathy. Although TO is associated with orbital expansion and occasional intraocular pressure elevations, these changes do not appear to translate into measurable alterations in LC curvature in the absence of dysthyroid optic neuropathy. This observation implies that LCCI may not serve as a sensitive structural biomarker for early or subclinical optic nerve involvement in stable or non-compressive disease. Nevertheless, LC curvature assessment may still be relevant in more advanced cases characterized by apical crowding or established compressive optic neuropathy, where sustained biomechanical stress is more likely to occur. Further longitudinal studies are warranted to determine whether progressive or severe forms of TO may induce detectable LC remodeling over time.

Several limitations should be acknowledged. First, the number of patients with active thyroid orbitopathy was relatively small, and age differed significantly among the study groups, with the active TO group being older than the inactive TO and control groups. Although the primary comparisons of LC parameters remained non-significant after adjustment for age and axial length, and after accounting for inter-eye correlation using GEE models, subgroup comparisons should still be interpreted with caution given the limited size of the active-TO subgroup. Second, no a priori sample-size calculation was performed, and post-hoc sensitivity analysis indicated that the study was underpowered (achieved power approximately 30–55%, depending on the assumed allocation, to detect a medium effect size of Cohen’s f = 0.25) to reliably exclude small-to-moderate between-group differences; the absence of statistically significant findings should therefore be interpreted as reflecting the precision limits of the present sample rather than as definitive evidence of no biological effect. Third, in a post-hoc, non-prespecified sensitivity analysis, LCT showed a nominally significant, unadjusted pairwise difference, being thinner in active TO compared with controls when both eyes were analyzed jointly with adjustment for age (*p* = 0.055); this observation was not corrected for multiple comparisons and requires confirmation in an adequately powered, prospective cohort before any biological interpretation is warranted. Fourth, intra-observer reproducibility of the manual LC measurements was not formally assessed in this study (e.g., via intraclass correlation coefficients on repeated measurements), which is a relevant limitation given that the principal finding is an absence of between-group differences; future studies should incorporate a formal reproducibility assessment. Fifth, LC parameters were obtained from a single horizontal B-scan through the center of the optic disc rather than from measurements averaged across multiple radial scans, which may not fully capture regional variation in LC morphology. Sixth, smoking status differed significantly among groups (most pronounced in the control group), and although not a central focus of this study, it represents an additional potential confounder of optic nerve head structure that could not be fully accounted for. Seventh, disease duration, prior IOP history, the numeric CAS score, and treatment history (e.g., immunosuppressive or anti-inflammatory therapy) were not systematically recorded in this study; IOP was measured only at a single time point (albeit concurrently with OCT imaging), precluding assessment of the duration or cumulative magnitude of pressure exposure, which may be more relevant to LC remodeling than a single measurement. Eighth, the cross-sectional design precludes assessment of longitudinal lamina cribrosa remodeling over the course of the disease. Finally, although swept-source OCT provides improved visualization of deep optic nerve head structures, lamina cribrosa measurements remain dependent on image quality and manual delineation, which may have introduced measurement variability.

## Conclusions

In this cross-sectional SS-OCT study, LCCI and other lamina cribrosa parameters were not significantly different between patients with thyroid orbitopathy without dysthyroid optic neuropathy and healthy controls. In this cohort, no statistically significant between-group difference in LCCI was detected according to either disease presence or clinical activity. Given that post-hoc sensitivity analysis indicated limited statistical power (approximately 30–55%) to detect a medium effect size in the present sample, these findings should be interpreted as reflecting the precision limits of this study rather than definitive evidence against a true biological effect, and they suggest that LCCI alone may have limited utility as a structural marker of disease presence or activity in uncomplicated thyroid orbitopathy. The relatively small number of patients with active disease, the resulting limited statistical power, the significant age difference among groups, the cross-sectional design, and single-center recruitment should be considered when interpreting these findings. Adequately powered, longitudinal studies with larger and age-balanced cohorts, formal assessment of measurement reproducibility, including patients at higher risk for dysthyroid optic neuropathy and with detailed orbital imaging, are needed to determine whether lamina cribrosa parameters may have a role in identifying eyes at risk for sight-threatening complications.

## Data Availability

The datasets used and/or analysed during the current study are available from the corresponding author on reasonable request.

## Declarations

### Generative AI and AI-assisted technologies in the writing process

During the preparation of this work, the authors used an AI-based language tool to improve the grammar, clarity, and readability of the English text. After using this tool, the authors reviewed and edited the content as needed and take full responsibility for the content of the published article.

## Abbreviations

AL: Axial length
BMO: Bruch’s membrane opening
CAS: Clinical activity score
DON: Dysthyroid optic neuropathy
HE: Hertel exophthalmometry
IOP: Intraocular pressure
LC: Lamina cribrosa
LCC: Lamina cribrosa curvature
LCCD: Lamina cribrosa curvature depth
LCCI: Lamina cribrosa curvature index
LCCW: Lamina cribrosa curvature width
LCD: Lamina cribrosa depth
LCT: Lamina cribrosa thickness
OCT: Optical coherence tomography
SS-OCTA: Swept-source optical coherence tomography angiography
TO: Thyroid orbitopathy
